# Therapeutic efficacy of chest ultrasound and chest x-ray after cardiac surgery

**DOI:** 10.1186/2197-425X-3-S1-A604

**Published:** 2015-10-01

**Authors:** A Vezzani, T Manca, F Corradi, C Brusasco, P Guido, F Benassi, F Nicolini, T Gherli

**Affiliations:** Department of Surgery, Cardiac Surgery Intensive Care Unit, Parma, Italy; Department of Critical Care, Intensive Care Unit, E.O. Ospedali Galliera, Genova, Italy; Department of Critical Care, University of Genova, Genova, Italy

## Introduction

Chest ultrasound (CU) has been suggested as an alternative to CXR to detect the majority of pulmonary abnormalities and misplacements of central venous lines. A good diagnostic accuracy has been reported for CU in intensive care units and postoperative setting, whereas chest auscultation (CA) shows a poorer diagnostic accuracy ([1], [2]) than CXR. The use of CA, CU and CXR as guides for treatment has been seldom reported ([3]).

## Objectives

The aim of this study was therefore to evaluate the usefulness of CA, CU and CXR to identify clinically significant findings in cardiac surgical patients and to measure their therapeutic efficacy.

## Methods

This study is a post-hoc analysis of a previous prospective observational study on the diagnostic value of CU after cardiac surgery ([1]). On admission to ICU, all patients had chest auscultation, ultrasound and chest x-ray. Any abnormality detected by each method and suggesting a change in clinical management was noted. For each method, the therapeutic efficacy was calculated as the ratio of number of exams indicating changes in management to the total number of exams. *k* statistics were used to assess the agreements of CU and CA with CXR.

## Results

Ninety-four of the 151 patients included (62%) showed abnormalities on chest X-ray. Chest ultrasound classified correctly 144 patients and chest auscultation 76. Abnormalities detected by chest X-ray requiring interventions were 16 (10%), one consolidation needing bronchoscopy, 7 alveolar-interstitial syndromes needing diuretic therapy, 2 pleural effusions and 2 pneumothoraxes needing drainage positioning, 2 endotracheal tube misplacements and 2 central venous catheter misplacements to be corrected. CXR was not able to identify 3 pericardial effusions. Number of intervention and therapeutic efficacy of each method are summarized in Table 1. The overall agreement for clinically significant interventions suggested by the CXR was very good for chest ultrasound and weak for chest auscultation.

**Table 1:** Therapeutic efficacy of each methods.

Type of intervention	Chest Ultrasound	Chest Auscultation	Chest x-Ray
Broncoscopy	1	0	1
Start of diuretic therapy	5	1	7
Pleural effusion drainage	2	0	2
Pneumothorax drainage	2	2	2
Pericardial effusion drainage	3	0	0
Change of endotracheal tube position	2	2	2
Central venous Catheter relocated	2	0	2
Therapeutic Efficacy	11% (17/151)	2,9% (5/151)	10% (16/151)

## Conclusions

although therapeutic efficacies of CU and CXR are relatively low, both methods are useful to identify clinically significant findings not discovered by CA in postoperative setting.
